# 2,2′-[4,10-Bis(carb­oxy­meth­yl)-4,10-diaza-1,7-diazo­niacyclo­dodecane-1,7-di­yl]diacetate dihydrate

**DOI:** 10.1107/S1600536811004843

**Published:** 2011-02-16

**Authors:** Paul S. Szalay, Matthias Zeller, Allen D. Hunter

**Affiliations:** a Department of Chemistry, Muskingum University, 163 Stormont St, New Concord, OH 43762, USA; b Department of Chemistry, Youngstown State University, One University Plaza, Youngstown, OH 44555, USA

## Abstract

In the title compound, C_16_H_28_N_4_O_8_·2H_2_O, the 12-membered macrocycle has twofold crystallographic symmetry and the asymmetric unit comprises one half-mol­ecule. The four carbox­yl/carboxyl­ate groups reside on the same side of the macrocycle. The mol­ecule is a double zwitterion with two of the carb­oxy­lic acid H atoms transferred to the two N atoms on the opposite sides of the macrocycle, resulting in both N atoms having positive charges and leaving the two resulting carboxyl­ate groups with negative charges. The two remaining carb­oxy­lic acid groups and the carboxyl­ate groups form O—H⋯O hydrogen bonds with the crystal water mol­ecules. The H atoms bound to the N atoms within the macrocyle are engaged in two equivalent hydrogen bonds with the adjacent N atoms.

## Related literature

Kumagai *et al.* (2002 ▶) describe different coordinations for carboxyl­ate groups. For background information about the title compound and its metal complexes, see: Viola-Villegas & Doyle (2009 ▶). For macrocycle configurations, see: Bosnich *et al.* (1965 ▶); Dale (1973 ▶, 1976 ▶, 1980 ▶); Meyer *et al.* (1998 ▶).
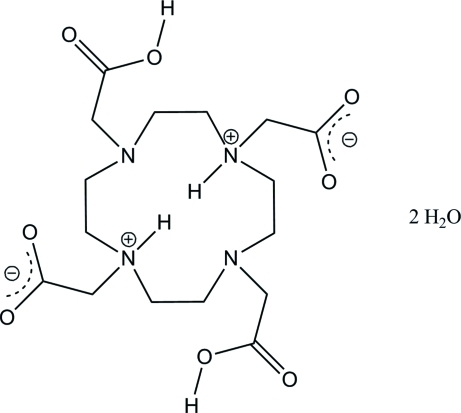


## Experimental

### 

#### Crystal data

C_16_H_28_N_4_O_8_·2H_2_O
*M* *_r_* = 440.46
Orthorhombic, 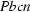

*a* = 17.183 (2) Å
*b* = 6.5826 (9) Å
*c* = 17.983 (2) Å
*V* = 2034.0 (5) Å^3^
*Z* = 4
Mo *K*α radiation
μ = 0.12 mm^−1^
*T* = 100 K
0.43 × 0.27 × 0.27 mm

#### Data collection

Bruker SMART APEX CCD diffractometer
Absorption correction: multi-scan (*SADABS*; Bruker 2003 ▶) *T*_min_ = 0.810, *T*_max_ = 1.000
19408 measured reflections
2520 independent reflections
2236 reflections with *I* > 2σ(*I*)
*R*_int_ = 0.037

#### Refinement

*R*[*F* ^2^ > 2σ(*F* ^2^)] = 0.042
*wR*(*F* ^2^) = 0.112
*S* = 1.08
2520 reflections
144 parameters
2 restraints
H atoms treated by a mixture of independent and constrained refinement
Δρ_max_ = 0.70 e Å^−3^
Δρ_min_ = −0.19 e Å^−3^

### 

Data collection: *SMART* (Bruker, 2001 ▶); cell refinement: *SAINT-Plus* (Bruker, 2001 ▶); data reduction: *SAINT-Plus*; program(s) used to solve structure: *SHELXS97* (Sheldrick, 2008 ▶); program(s) used to refine structure: *SHELXL97* (Sheldrick, 2008 ▶); molecular graphics: *SHELXTL* (Sheldrick, 2008 ▶); software used to prepare material for publication: *SHELXL97* and *PLATON* Spek (2009) ▶.

## Supplementary Material

Crystal structure: contains datablocks global, I. DOI: 10.1107/S1600536811004843/kp2290sup1.cif

Structure factors: contains datablocks I. DOI: 10.1107/S1600536811004843/kp2290Isup2.hkl

Additional supplementary materials:  crystallographic information; 3D view; checkCIF report

**Fig. 3.**
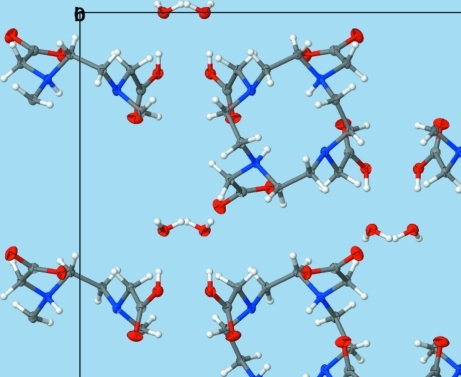
Enhanced figure view of the packing of the title compound along the b axis.

Enhanced figure: interactive version of Fig. 3

## Figures and Tables

**Table 1:** Hydrogen-bond geometry (Å, °)

*D*—H⋯*A*	*D*—H	H⋯*A*	*D*⋯*A*	*D*—H⋯*A*
O3—H3⋯O5	0.84	1.71	2.5295 (14)	166
N1—H1⋯N2	0.93	2.44	2.8940 (14)	110
O5—H5*D*⋯O1^i^	0.86 (1)	1.78 (2)	2.6380 (14)	173 (2)
O5—H5*C*⋯O1^ii^	0.84 (1)	1.85 (2)	2.6776 (14)	170 (2)

Symmetry codes: (i) 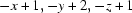; (ii) 
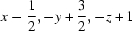
.

**Fig. 1.**
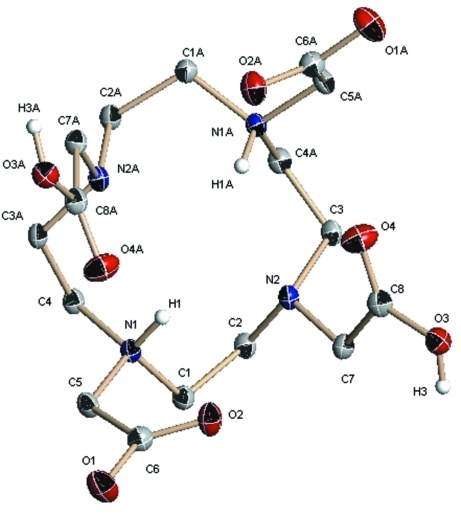
The structure of the title compound [DOTAH4] and water molecule (hydrogen atoms bound to carbon atoms are omitted for clarity). Dispalacement ellipsoids are shown at the 50% probability level. The two fold rotation axis that generates the symmetry related half of the molecule has a site symmetry of 4c.

**Fig. 2.**
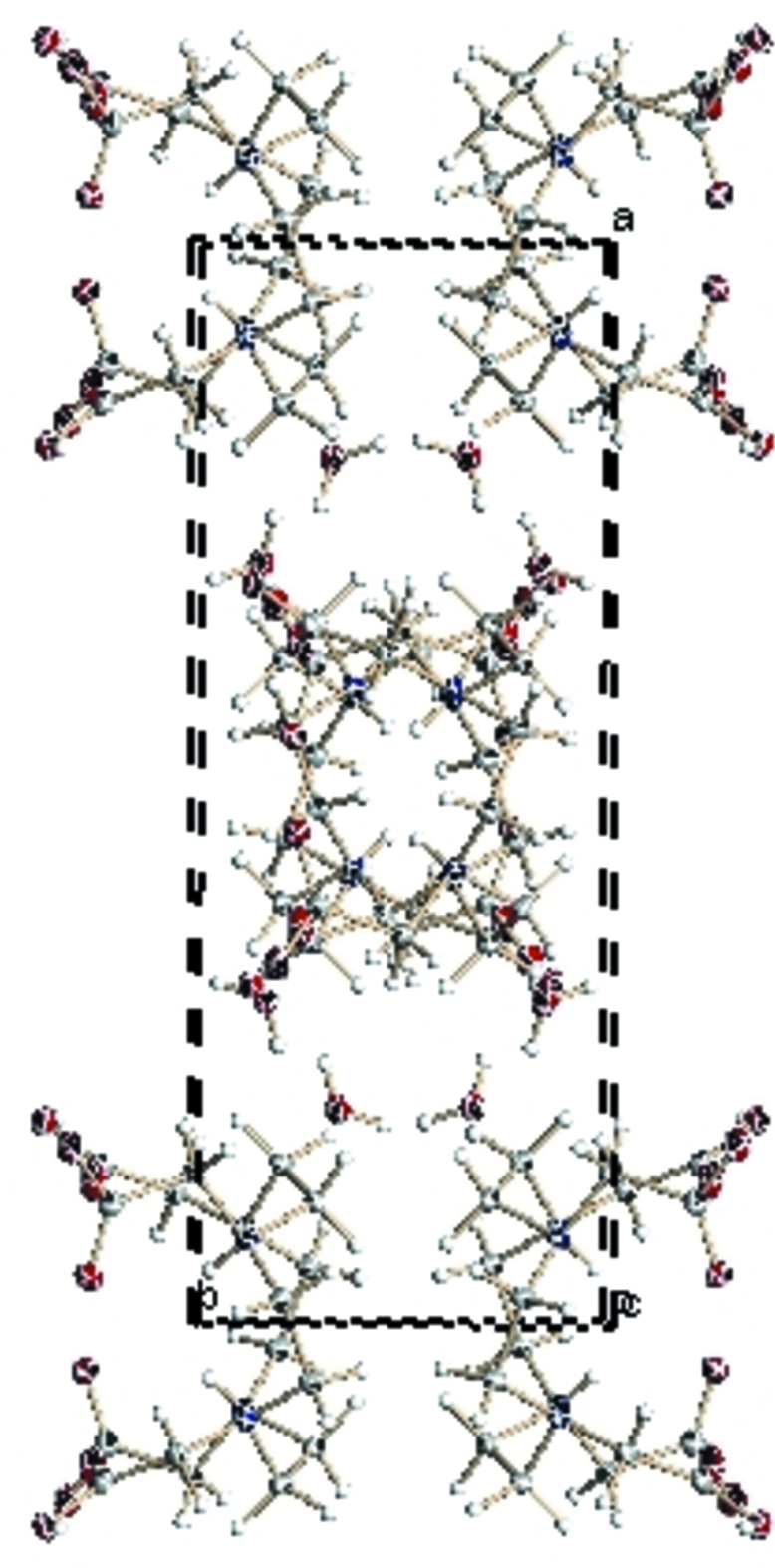
A packing diagram of [DOTAH4] as viewed along the c axis.

## supplementary crystallographic information

### Comment

In the course of our studies to prepare coordination polymer and metal-organic framework type compounds we investigated the title compound as a potentional building block. The molecule 1,4,7,10-tetraazacyclodecane-1,4,7,10-tetraacetic acid DOTAH_4_ and its deprotonated analogs, [DOTAH_2_]^2-^ and [DOTA]^4-^ have several features desirable in coordination chemistry. The ligand offers a macrocycle with four neutral nitrogen donor sites as well as four dangling carboxylic acid groups. Carboxylate groups when deprotonated have been shown to exhibit nine different coordination modes with metal ions, seven of which coordinate two or more metal ions (Kumagai *et al.*, 2002). Therefore, the potential for forming molecular species as well as coordination polymers or metal-organic framework type compounds exists for this organic building block. For numerous examples of metal containing DOTAH_4_ compounds and its charged analogues see Viola-Villegas & Doyle (2009).

Only half of DOTAH_4_ molecule (Fig. 1) in the structure is an asymmetric unit. The other half of the macrocycle is generated by a twofold rotation axis parallel to the *b* axis. There is no significant ring strain based on an analysis of the bond angles withing the ring. The ring is composed of eight methylene carbons (C1—C4, C1^i^—C4^i^), two ammonium N atoms (N1, N1^i^) and two tertiary N atoms (N2, N2^i^) (symmetry operator (i): -*x* + 1, *y*, -*z* + 3/2). The bond angles between them range from 110–112°. The configuration has all four N atoms located above the eight methylene carbons along the direction of the twofold axis in the centre of the ring producing a basket-like shape that would be able to coordinate a metal without large changes of the overall structure of the molecule. According to the system outlined by Dale this arrangement would be described as (3,3,3,3)-B (Dale, 1973, 1976, 1980, Meyer *et al.*, 1998). This system uses numbers to indicate the number of chemical bonds between the genuinie corners in the macrocycle. Genuine corners are the central atoms in an anti-*gauche*-*gauche*-anti bond sequence. In the title compound the atoms C1, C3, C1A, and C3A constitute genuine corners which are separated from each other along the macrocycle by three bonds. The "B" designation indicates that the four heteroatoms in this 12-membered macrocylce reside in a square planar arrangement above the methylene carbons (as described above). Using the terminology of Bosnich *et al.* (1965) the configuration of the macrocycle would be *cis*-I since all of the carboxylate containing groups project in the same direction.

The weighted average ring bond distance is 1.503 Å (*PLATON*, Spek (2009)). The weighted average absolute torsion angle is 100.45°. The total puckering amplitude of the ring is 1.526 Å. The four carboxylic acid and carboxylate groups are bound to the N atoms and also all reside above the macrocycle. This arrangement leads to well separated hydrophobic and hydrophilic parts within the molecule. The H atoms bound to the N atoms within the macrocyle are engaged in two equivalent hydrogen bonds with the adjacent nitrogen atoms (N1—H1···N2, N1^i^—H1^i^···N2^i^, Table 1). The N1—H1 and H1···N2 distances are 0.93 Å and 2.44 Å respectively. The angle between the donor and acceptor is 110.1° in accord with the donor and acceptor both residing within the ring and being separated by two atoms.

The crystal packing (Figs. 2 and 3) is dominated by hydrogen bonding between the crystal water molecules and the carboxylic acid and carboxylate groups. Each water molecule forms three hydrogen bonding interactions with the two hydrogen atoms oriented towards carboxylate groups (O5—H5D···O1^ii^, O5—H5C···O1^iii^, Table 1) and the oxygen directed towards a carboxylic acid group (O3—H3···O5, Table 1).

### Experimental

The title compound 1,4,7,10-tetraazacyclodecane-1,4,7,10-tetraacetic acid was purchased from Strem Chemicals and used without further purification.

The compound was crystallized from a saturated DMSO solution. A DMSO solution (2 mL) was saturated with DOTAH_4_ at 323 K. Upon cooling to room temperature and sitting for four days colourless block shaped crystals were formed.

### Refinement

The oxygen to hydrogen bond distances in the solvent water molecule were restrained to be 0.84 Å with a standard deviation of 0.02 Å. They were set to have an isotropic displacement parameter of 1.5 times that of the adjacent oxygen atom. The same displacement parameter was used for the hydrogen bound to the carboxylic acid, which were placed in calculated positions at a distance of 0.84 Å from the O atom but that were allowed to freely rotate at a fixed angle around the C—O bond to best fit the experimental electron density. All other hydrogen atoms in the structure were placed in calculated positions with *X*—H distances of 0.99 (methylene) or 0.93 Å (amine) with *U*_iso_(H) = 1.2 *U*_eq_(*X*).

The highest residual electron density peak in the final Fourier map, with a heigth of 0.70 e^-^×Å^-3^, is located at the center of the macrocylce. An electron density difference Fourier map cutting through the protonated amine N atoms and the center of the residual electron density in the middle of the ring (with the protic amine H atoms removed prior to generation of the map) shows electron densities in the positions of the amine H atoms that are substantially larger than that of the residual electron density in the center of the ring, thus indicating that the amine H atoms are indeed fully protonated (which is supported by a refinement of the amine H atom occupancy, which yielded full occupancy). The residual density in the center of the ring refines to about 60% of one electron and it is located on a special position (site symmetry of 4c).

### Crystal data

**Table:**

C_16_H_28_N_4_O_8_·2H_2_O	*D*_x_ = 1.438 Mg m^−^^3^
*M**_r_* = 440.46	Mo *K*α radiation, λ = 0.71073 Å
Orthorhombic, *P**b**c**n*	Cell parameters from 13988 reflections
*a* = 17.183 (2) Å	θ = 1.0–28.3°
*b* = 6.5826 (9) Å	µ = 0.12 mm^−^^1^
*c* = 17.983 (2) Å	*T* = 100 K
*V* = 2034.0 (5) Å^3^	Block, colourless
*Z* = 4	0.43 × 0.27 × 0.27 mm
*F*(000) = 944	

### Data collection

**Table:**

Bruker SMART APEX CCD diffractometer	2520 independent reflections
Radiation source: fine-focus sealed tube	2236 reflections with *I* > 2σ(*I*)
graphite	*R*_int_ = 0.037
ω scans	θ_max_ = 28.3°, θ_min_ = 2.3°
Absorption correction: multi-scan (*SADABS*; Bruker 2003)	*h* = −22→22
*T*_min_ = 0.810, *T*_max_ = 1.000	*k* = −8→8
19408 measured reflections	*l* = −23→23

### Refinement

**Table:**

Refinement on *F*^2^	Primary atom site location: structure-invariant direct methods
Least-squares matrix: full	Secondary atom site location: difference Fourier map
*R*[*F*^2^ > 2σ(*F*^2^)] = 0.042	Hydrogen site location: inferred from neighbouring sites
*wR*(*F*^2^) = 0.112	H atoms treated by a mixture of independent and constrained refinement
*S* = 1.08	*w* = 1/[σ^2^(*F*_o_^2^) + (0.0616*P*)^2^ + 0.8651*P*] where *P* = (*F*_o_^2^ + 2*F*_c_^2^)/3
2520 reflections	(Δ/σ)_max_ = 0.003
144 parameters	Δρ_max_ = 0.70 e Å^−^^3^
2 restraints	Δρ_min_ = −0.19 e Å^−^^3^

### Special details

**Table:**

Geometry. All e.s.d.'s (except the e.s.d. in the dihedral angle between two l.s. planes) are estimated using the full covariance matrix. The cell e.s.d.'s are taken into account individually in the estimation of e.s.d.'s in distances, angles and torsion angles; correlations between e.s.d.'s in cell parameters are only used when they are defined by crystal symmetry. An approximate (isotropic) treatment of cell e.s.d.'s is used for estimating e.s.d.'s involving l.s. planes.
Refinement. Refinement of *F*^2^ against ALL reflections. The weighted *R*-factor *wR* and goodness of fit *S* are based on *F*^2^, conventional *R*-factors *R* are based on *F*, with *F* set to zero for negative *F*^2^. The threshold expression of *F*^2^ > σ(*F*^2^) is used only for calculating *R*-factors(gt) *etc*. and is not relevant to the choice of reflections for refinement. *R*-factors based on *F*^2^ are statistically about twice as large as those based on *F*, and *R*- factors based on ALL data will be even larger.

### Fractional atomic coordinates and isotropic or equivalent isotropic displacement parameters (Å^2^)

**Table:**

	*x*	*y*	*z*	*U*_iso_*/*U*_eq_	
C1	0.52319 (7)	0.29809 (18)	0.59684 (7)	0.0172 (2)	
H1A	0.5484	0.1817	0.5716	0.021*	
H1B	0.5118	0.4028	0.5588	0.021*	
C2	0.44727 (7)	0.22770 (18)	0.63205 (7)	0.0169 (2)	
H2A	0.4101	0.1899	0.5923	0.020*	
H2B	0.4573	0.1052	0.6625	0.020*	
C3	0.35164 (7)	0.29561 (18)	0.72810 (7)	0.0161 (2)	
H3A	0.3257	0.1827	0.7015	0.019*	
H3B	0.3118	0.3994	0.7398	0.019*	
C4	0.61331 (7)	0.21651 (18)	0.70032 (7)	0.0165 (2)	
H4A	0.6542	0.1467	0.6711	0.020*	
H4B	0.5724	0.1157	0.7123	0.020*	
C5	0.64046 (7)	0.50850 (18)	0.61660 (7)	0.0175 (2)	
H5A	0.6619	0.4297	0.5744	0.021*	
H5B	0.6833	0.5319	0.6524	0.021*	
C6	0.61183 (7)	0.71385 (19)	0.58773 (7)	0.0190 (3)	
C7	0.37872 (7)	0.54695 (18)	0.63298 (7)	0.0167 (2)	
H7A	0.3332	0.4922	0.6058	0.020*	
H7B	0.4178	0.5900	0.5957	0.020*	
C8	0.35349 (7)	0.73022 (18)	0.67787 (7)	0.0187 (3)	
N1	0.57832 (6)	0.38475 (15)	0.65388 (5)	0.0147 (2)	
H1	0.5502	0.4699	0.6852	0.018*	
N2	0.41201 (5)	0.38638 (15)	0.67930 (6)	0.0156 (2)	
O1	0.66535 (6)	0.81050 (15)	0.55408 (6)	0.0293 (2)	
O2	0.54404 (5)	0.76758 (14)	0.59796 (6)	0.0248 (2)	
O3	0.31304 (5)	0.86834 (13)	0.64068 (5)	0.0201 (2)	
H3	0.3110	0.8356	0.5956	0.030*	
O4	0.36798 (7)	0.75428 (15)	0.74325 (5)	0.0286 (2)	
O5	0.29964 (6)	0.82984 (15)	0.50131 (5)	0.0218 (2)	
H5C	0.2571 (9)	0.800 (3)	0.4810 (9)	0.033*	
H5D	0.3139 (11)	0.942 (2)	0.4806 (10)	0.033*	

### Atomic displacement parameters (Å^2^)

**Table:**

	*U*^11^	*U*^22^	*U*^33^	*U*^12^	*U*^13^	*U*^23^
C1	0.0153 (5)	0.0185 (5)	0.0176 (5)	−0.0004 (4)	−0.0003 (4)	−0.0007 (4)
C2	0.0144 (5)	0.0160 (5)	0.0204 (6)	−0.0006 (4)	−0.0001 (4)	−0.0015 (4)
C3	0.0124 (5)	0.0153 (5)	0.0205 (6)	−0.0014 (4)	−0.0004 (4)	−0.0014 (4)
C4	0.0159 (5)	0.0127 (5)	0.0209 (6)	0.0024 (4)	−0.0001 (4)	0.0018 (4)
C5	0.0141 (5)	0.0164 (5)	0.0219 (6)	−0.0006 (4)	0.0033 (4)	0.0026 (4)
C6	0.0210 (6)	0.0160 (5)	0.0201 (6)	−0.0001 (4)	−0.0001 (4)	0.0014 (4)
C7	0.0148 (5)	0.0157 (5)	0.0195 (5)	0.0005 (4)	−0.0017 (4)	0.0005 (4)
C8	0.0183 (6)	0.0143 (5)	0.0236 (6)	−0.0037 (4)	0.0004 (4)	0.0004 (4)
N1	0.0132 (4)	0.0137 (5)	0.0173 (5)	0.0007 (3)	0.0010 (3)	0.0008 (4)
N2	0.0127 (4)	0.0142 (5)	0.0198 (5)	0.0004 (3)	0.0003 (4)	0.0010 (4)
O1	0.0287 (5)	0.0212 (5)	0.0381 (6)	0.0002 (4)	0.0111 (4)	0.0102 (4)
O2	0.0187 (5)	0.0196 (4)	0.0361 (5)	0.0025 (4)	−0.0007 (4)	0.0035 (4)
O3	0.0218 (4)	0.0158 (4)	0.0227 (4)	0.0021 (3)	0.0013 (3)	−0.0007 (3)
O4	0.0445 (6)	0.0186 (5)	0.0227 (5)	−0.0021 (4)	−0.0053 (4)	−0.0026 (4)
O5	0.0234 (5)	0.0173 (4)	0.0246 (5)	0.0009 (4)	−0.0023 (4)	0.0023 (3)

### Geometric parameters (Å, °)

**Table:**

C1—N1	1.5082 (15)	C5—C6	1.5294 (17)
C1—C2	1.5223 (16)	C5—H5A	0.9900
C1—H1A	0.9900	C5—H5B	0.9900
C1—H1B	0.9900	C6—O2	1.2312 (16)
C2—N2	1.4765 (15)	C6—O1	1.2715 (16)
C2—H2A	0.9900	C7—N2	1.4622 (15)
C2—H2B	0.9900	C7—C8	1.5149 (17)
C3—N2	1.4844 (15)	C7—H7A	0.9900
C3—C4^i^	1.5136 (16)	C7—H7B	0.9900
C3—H3A	0.9900	C8—O4	1.2122 (16)
C3—H3B	0.9900	C8—O3	1.3255 (15)
C4—N1	1.5117 (15)	N1—H1	0.9300
C4—C3^i^	1.5136 (17)	O3—H3	0.8400
C4—H4A	0.9900	O5—H5C	0.840 (15)
C4—H4B	0.9900	O5—H5D	0.865 (15)
C5—N1	1.5011 (15)		
			
N1—C1—C2	111.75 (10)	N1—C5—H5B	108.8
N1—C1—H1A	109.3	C6—C5—H5B	108.8
C2—C1—H1A	109.3	H5A—C5—H5B	107.7
N1—C1—H1B	109.3	O2—C6—O1	127.71 (12)
C2—C1—H1B	109.3	O2—C6—C5	120.50 (11)
H1A—C1—H1B	107.9	O1—C6—C5	111.78 (11)
N2—C2—C1	112.06 (10)	N2—C7—C8	112.59 (10)
N2—C2—H2A	109.2	N2—C7—H7A	109.1
C1—C2—H2A	109.2	C8—C7—H7A	109.1
N2—C2—H2B	109.2	N2—C7—H7B	109.1
C1—C2—H2B	109.2	C8—C7—H7B	109.1
H2A—C2—H2B	107.9	H7A—C7—H7B	107.8
N2—C3—C4^i^	111.30 (9)	O4—C8—O3	120.49 (12)
N2—C3—H3A	109.4	O4—C8—C7	124.21 (12)
C4^i^—C3—H3A	109.4	O3—C8—C7	115.30 (11)
N2—C3—H3B	109.4	C5—N1—C1	110.39 (9)
C4^i^—C3—H3B	109.4	C5—N1—C4	111.18 (9)
H3A—C3—H3B	108.0	C1—N1—C4	110.39 (9)
N1—C4—C3^i^	112.09 (9)	C5—N1—H1	108.3
N1—C4—H4A	109.2	C1—N1—H1	108.3
C3^i^—C4—H4A	109.2	C4—N1—H1	108.3
N1—C4—H4B	109.2	C7—N2—C2	110.13 (9)
C3^i^—C4—H4B	109.2	C7—N2—C3	110.75 (9)
H4A—C4—H4B	107.9	C2—N2—C3	110.02 (9)
N1—C5—C6	113.73 (10)	C8—O3—H3	109.5
N1—C5—H5A	108.8	H5C—O5—H5D	105.1 (18)
C6—C5—H5A	108.8		
			
N1—C1—C2—N2	51.11 (13)	C3^i^—C4—N1—C5	74.84 (12)
N1—C5—C6—O2	2.30 (17)	C3^i^—C4—N1—C1	−162.30 (9)
N1—C5—C6—O1	−177.48 (11)	C8—C7—N2—C2	−170.60 (9)
N2—C7—C8—O4	8.99 (17)	C8—C7—N2—C3	67.48 (12)
N2—C7—C8—O3	−170.88 (10)	C1—C2—N2—C7	73.24 (12)
C6—C5—N1—C1	73.84 (12)	C1—C2—N2—C3	−164.40 (10)
C6—C5—N1—C4	−163.30 (10)	C4^i^—C3—N2—C7	−150.74 (10)
C2—C1—N1—C5	−162.75 (9)	C4^i^—C3—N2—C2	87.27 (11)
C2—C1—N1—C4	73.93 (12)		

Symmetry codes: (i) −*x*+1, *y*, −*z*+3/2.

### Hydrogen-bond geometry (Å, °)

**Table:**

*D*—H···*A*	*D*—H	H···*A*	*D*···*A*	*D*—H···*A*
O3—H3···O5	0.84	1.71	2.5295 (14)	166
N1—H1···N2	0.93	2.44	2.8940 (14)	110
O5—H5D···O1^ii^	0.86 (1)	1.78 (2)	2.6380 (14)	173.(2)
O5—H5C···O1^iii^	0.84 (1)	1.85 (2)	2.6776 (14)	170.(2)

Symmetry codes: (ii) −*x*+1, −*y*+2, −*z*+1; (iii) *x*−1/2, −*y*+3/2, −*z*+1.
